# Massive Acetaminophen Overdose Treated Successfully with N-Acetylcysteine, Fomepizole, and Hemodialysis

**DOI:** 10.1155/2021/6695967

**Published:** 2021-07-11

**Authors:** Michael H. Chiu, Natalia Jaworska, Nicholas L. Li, Mark Yarema

**Affiliations:** ^1^Department of Critical Care Medicine, Cumming School of Medicine, University of Calgary, AB, Canada; ^2^Department of Medicine, Division of Nephrology, Cumming School of Medicine, University of Calgary, AB, Canada; ^3^Poison and Drug Information Service, Alberta Health Services, Calgary, AB, Canada; ^4^Department of Emergency Medicine, University of Calgary, Calgary, AB, Canada

## Abstract

Acetaminophen overdose is one of the most common causes of acute hepatic failure in the developed world. There is strong evidence for N-acetylcysteine (NAC) as a safe and effective antidote for acetaminophen toxicity. However, there is less clarity in the management of massive overdoses (acute, single ingestions > 500 mg/kg with 4-hour equivalent concentrations ~6000 *μ*mol/L) which are often associated with metabolic acidosis and multiorgan dysfunction. In such ingestions, the role of adjuvant treatments such as fomepizole and extracorporeal removal is unclear. We present a case of a 20-year-old female presenting with an acute ingestion of over 120 grams (1764.7 mg/kg) and an acetaminophen concentration of 5880 *μ*mol/L who developed refractory shock, decreased level of consciousness, and metabolic acidosis requiring mechanical ventilation and vasopressor support. She was treated with gastric decontamination with activated charcoal, IV NAC, fomepizole, and hemodialysis. The patient had complete clearance of acetaminophen by 32 hours after presentation and normalization of her acid base and hemodynamic status without any organ failure. This case highlights the potential benefit of a triple strategy of NAC, fomepizole, and early hemodialysis in massive acetaminophen overdose, potentially sparing complications of prolonged intubation and ICU hospitalization.

## 1. Introduction

Acetaminophen (APAP) toxicity is among the most common medication-related overdoses and accounts for nearly half of all liver transplantations in the United States [1]. Poisoning can occur through single or repeated ingestions. With therapeutic dosing, APAP is metabolized primarily via glucuronidation and sulfation to nontoxic metabolites. With supratherapeutic ingestions, up to 50% of absorbed APAP is metabolized by CYP2E1, leading to production of N-acetyl-p-benzoquinone imine (NAPQI), the hepatotoxic metabolite. Management consists of gastric decontamination and early administration of N-acetylcysteine (NAC) if the [APAP] is potentially toxic when plotted on the Rumack-Matthew acetaminophen treatment nomogram. NAC has multiple therapeutic actions including acting as both a glutathione precursor and substitute, enhancing the nontoxic sulfation metabolic pathway (thereby reducing production of NAPQI, improving of hepatic blood flow, and scavenging of free radicals) [1]. Clinical manifestations of APAP poisoning are typically nonspecific however can be divided into four stages. Stage 1 occurs within 24 hours and is characterized by nausea, vomiting, lethargy, and malaise. Stage 2 (24 to 27 hours) involves improvement in nausea and vomiting, with development of right upper quadrant pain, and worsening hepatic and renal toxicity. In stage 3 (72-96 hours), liver function abnormalities peak. Depending on the degree of poisoning, hepatic failure with jaundice, hepatic encephalopathy, and bleeding diathesis occur. Development of multiorgan failure and death commonly occur in this phase. Patients who survive stage 3 progress to stage 4 with a slow recovery and normalization of liver chemistry (4 days to two weeks) [2].

In massive overdoses (above 500 mg/kg), clinical deterioration may occur rapidly (within 12 hours) with decreased level of consciousness and mitochondrial injury characterized by a high anion gap metabolic acidosis preceding severe liver injury [3]. In such cases, protective effects of NAC may be overwhelmed by the ingested dose. There is less evidence surrounding the management of massive overdoses; however, the literature is supportive of the use of additional therapies [3]. Novel adjuvant therapies include the addition of fomepizole via inhibition of CYP2E1 and extracorporeal removal. Currently, there is no consensus on when to initiate these therapies.

Massive overdose may require treatment with IV NAC beyond 21 hours and may expose the patient to prolonged intubation and ICU admission with potential complications of hospital-acquired infections and neuromuscular weakness. There are theoretical benefits in rapid acetaminophen elimination and early correction of metabolic acidosis; however, this remains an area of ongoing research. We report a case of massive acetaminophen overdose successfully treated with early administration of intravenous NAC, fomepizole, and prolonged intermittent hemodialysis.

## 2. Case Report

A 20-year-old female weighing 68 kg, with anxiety and a history of self-harm and prior suicide attempts, presented to hospital after being found after an acute ingestion of approximately 120 g of acetaminophen, 65 g of ibuprofen, and 300 mg of oxycodone. She presented to emergency department one-hour postingestion. Her oxygen saturation was 96% on room air with a respiratory rate of 20 breaths per minute, and an initial Glasgow Coma Scale was 13. She was tachycardic with a heart rate of 130 beats per minute with hypotension with a blood pressure of 72/45. Her clinical exam was remarkable for symmetrical and pinpoint pupils, mild abdominal tenderness, and previous evidence of self-harm.

Arterial blood gas revealed a metabolic acidosis with a pH of 6.89, PCO2 26 mmHg, bicarbonate of 5 mM, lactate of 13.8 mM, and methemoglobin concentration of 11.6%. Serum blood work showed a leukocytosis at 15.6 × 10^9^ cells/L and hyponatremia and hypokalemia (134 mM and 3.3 mM, respectively) with a HCO3 content of 11 mM and creatinine 21 *μ*mol/L. Acetaminophen concentration was 5880 *μ*mol/L two and a half hours postingestion with liver chemistry ALT 16 U/L, ALP 63 U/L, GGT 7 U/L, total bilirubin 11 *μ*mol/L, and an INR of 1.4. Salicylate concentration was <0.07 mmol/L with an ethanol level of <2 mM. Electrocardiogram demonstrated sinus tachycardia, and the chest X-ray was normal. CT of the head revealed no concerning pathology and no evidence of cerebral edema. The patient had hyperdynamic biventricular function on echocardiogram.

**Figure 1:**
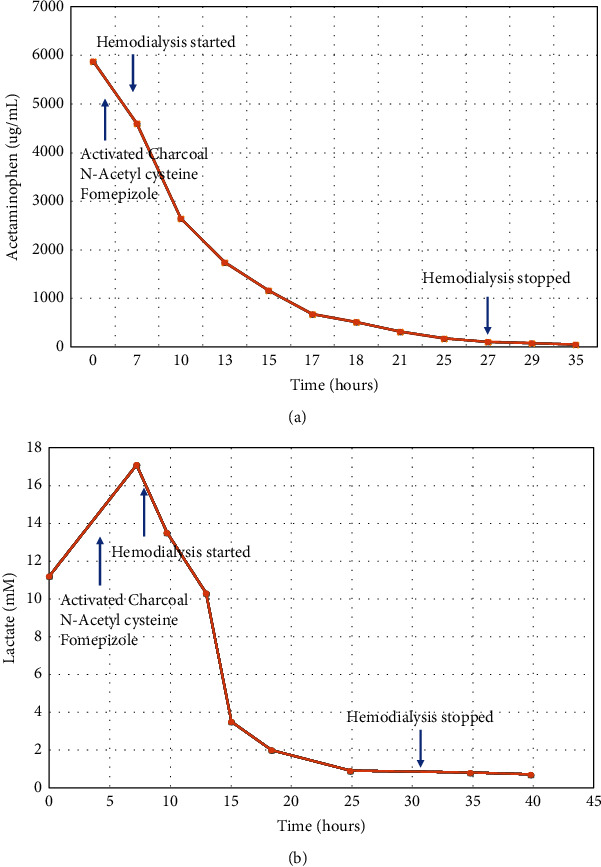
(a) Acetaminophen concentrations versus time with timing of treatment for acetaminophen toxicity. (b) Lactate concentrations versus time.

Her GCS decreased rapidly from 13 to 3, for which she was intubated. She was treated with norepinephrine 0.3 mcg/kg/min and epinephrine 0.2 mcg/kg/min and was given intravenous sodium bicarbonate. The medical toxicology service was consulted and IV N-acetylcysteine (150 mg/kg loading dose over 1 hour then 15 mg/kg/hr for 3 days) and fomepizole (1000 mg) were administered approximately three hours postingestion. The patient was admitted to ICU and started on intermittent hemodialysis approximately six hours postingestion. Hemodialysis was pursued given the patient's metabolic acidosis, decreased level of consciousness, markedly elevated acetaminophen concentration, and high lactate. Dialysis was performed with a Toray Filtryzer BG-2.1U (Japan) dialyzer (Qb 300 ml/min and a Qd of 500 mL/min) with heparin at 500 U/hr for anticoagulation. No ultrafiltration was performed. The dialysate had a sodium concentration of 134 mM, potassium 4.0 mM, HCO3 38 mM, and calcium 1.25 mM. Serum potassium required continuous IV replacement, in addition to high dialysate potassium, due to shifting with correction of metabolic acidosis. Intermittent hemodialysis and vasoactive medications were discontinued approximately 24 and 27 hours postingestion, respectively, with normalization of acid and base status and an [APAP] below 300 *μ*mol/L (Figure 1). There were no dialysis-associated complications aside from transient hypophosphatemia due to clearance from extended therapy. On day 2, her ALT peaked at 76 U/L, and INR peaked at 1.7.

She was maintained on IV NAC at 15 mg/kg/hr for 3 days until normalization of the INR and ALT. Fomepizole was given at a dose of 10 mg/kg every four hours during dialysis and for an additional day at 700 mg q12 h. In total, the patient received 3 days of IV NAC and 2 days of fomepizole. She was alert, oriented, and following commands at 24 hours postadmission and successfully extubated on her second day of admission. She was transferred to the inpatient psychiatry service in stable condition after a 3-day stay in the intensive care unit.

## 3. Discussion

We present a case of a 20-year-old female with a polypharmacy ingestion of acetaminophen and ibuprofen who developed refractory shock, decreased level of consciousness, and severe metabolic acidosis requiring mechanical ventilation and vasopressor support. The clinical presentation of our patient is consistent with what has been previously described in the setting of massive acetaminophen ingestions defined as a single ingestion of acetaminophen of greater than 500 mg/kg^3^. Our patient presented with the characteristic findings of massive acetaminophen ingestion including the presence of altered level of consciousness and early metabolic acidosis (within 8 hours of ingestion) with an elevated lactate concentration in the absence of hepatic injury [4]. Methemoglobinemia, as was seen in our case, has also been described in massive acetaminophen ingestions and may occur as a result of increased oxidative stress from high [APAP] exposure [4].

N-acetylcysteine (NAC) has been an established therapy for prevention of acetaminophen hepatotoxicity since the 1970s [5, 6]. While a majority of cases of acetaminophen toxicity recover with NAC treatment alone, in massive acetaminophen ingestions, NAC may be insufficient [7]. Several case reports have highlighted the development of acute liver injury and hepatotoxicity despite appropriate treatment with NAC in the setting of massive acetaminophen ingestions [8–10]. In one retrospective cohort study of 727 admissions for acute APAP toxicity where ingestions occurred over less than 2 hours and intravenous NAC was administered within 24 h of ingestion, patients with higher concentrations of APAP at hospital presentation developed acute liver injury (ALT activity > 150 U/L and double admission value) and hepatotoxicity (peak ALT activity > 1000 U/L) in a higher proportion than those patients with lower initial APAP concentrations despite appropriate treatment with standard 21-hour intravenous NAC infusion regimens [9]. Additional therapeutic interventions such as fomepizole and extracorporeal removal have been utilized in the treatment of massive acetaminophen ingestions [11–13].

4-Methylpyrazole, also known as fomepizole, is an alcohol dehydrogenase competitive antagonist [14] and a potent inhibitor of CYP2E1, the major isozyme in acetaminophen metabolism to NAPQI [15]. In addition to the inhibition of CYP2E1, fomepizole inhibits the c-jun N-terminal kinase (JNK) pathway responsible for mitochondrial dysfunction [16, 17]. In vitro studies with human hepatocytes and in vivo murine models have shown that cotreatment with fomepizole and acetaminophen has hepatoprotective effects with reduced plasma alanine aminotransferase concentrations and decreased hepatocyte necrosis [15]. Kang et al. recruited five healthy volunteers in a crossover trial examining the inhibition of CYP2E1 metabolism of acetaminophen following administration of a single supratherapeutic 80 mg/kg oral dose of APAP with fomepizole 15 mg/kg [18]. Decreased concentrations of urine and plasma oxidative metabolites of acetaminophen were found among all participants administered fomepizole [18]. Adverse effects of fomepizole have been reported with one postmarketing survey reporting 50 adverse reactions attributed to 36 patients from a total of 536 patients (7%) receiving fomepizole on initial suspicion of toxic alcohol ingestion [19]. The most commonly reported adverse reactions were local infusion site reactions and asymptomatic elevations in serum aminotransferases with no reported discontinuation of fomepizole as a result of these adverse reactions [19]. Widespread clinical data regarding the efficacy of fomepizole in acetaminophen toxicity remains sparse. In a series of six cases treated with fomepizole due to persistently elevated acetaminophen concentration levels at levels at risk of developing hepatic injury, Rampon et al. documented the successful management of six pediatric and adult patients treated with a combination of NAC therapy and fomepizole with no hepatic injury [20]. The decision to use fomepizole in these patients was because of the persistently elevated [APAP] despite intravenous NAC treatment. In our case, the patient was administered fomepizole and NAC early after their acetaminophen ingestion given the clinical severity on presentation. Despite rapid intervention, there was progression of clinical deterioration as a result of refractory metabolic acidosis necessitating the extracorporeal removal of acetaminophen.

The physical chemical and toxicokinetic properties of acetaminophen make it amenable to removal by intermittent hemodialysis, in particular its low molecular weight of 151 Da, low protein binding, and small volume of distribution of 0.9-1.0 L/kg^3^. The Extracorporeal Treatments in Poisoning (EXTRIP) workgroup has established guidelines for the indications of hemodialysis in the setting of massive acetaminophen ingestion [3]. Current recommendations from the EXTRIP workgroup suggest extracorporeal removal in the setting of severe acetaminophen poisoning, defined as [APAP] greater than 5960 *μ*mol/L with NAC administration, an altered level of consciousness, metabolic acidosis, and elevated lactate concentration [3].

What remains unclear is whether lower [APAP] thresholds for hemodialysis initiation result in sufficient acetaminophen clearance to affect clinical outcomes. NAC repletes glutathione stores and scavenges reactive oxygen species and remains the cornerstone of clinical management of acetaminophen toxicity; thus, the consequences of NAC clearance through hemodialysis must be considered. Ghannoum et al. reported the successful management of a case of an 18-year-old female presenting with early mitochondrial dysfunction from acetaminophen toxicity requiring hemodialysis. In their case, they report high dialyzability of NAC with a drug clearance of 190.3 ml/min and a total of 17.9 g of NAC removed through the hemodialysis circuit [21]. Previous literature suggests that up to 50% of NAC undergoes clearance with intermittent hemodialysis [22]. Experience with fomepizole in ethylene glycol poisoning patients has also shown that fomepizole is vulnerable to increased clearance via hemodialysis with up to 45% of fomepizole eliminated in dialysate in two reported cases [23]. Currently, there are no specific dosing adjustment guideline recommendations in the setting of intermittent hemodialysis for either fomepizole or NAC. Our patient was managed with an NAC infusion dosing of 15 mg/kg/hr to account for the total amount of NAC predicted to be removed by hemodialysis, as well as an increase in frequency of fomepizole dosing to every 4 hours to account for that removed by dialysis.

Our patient also required potassium replacement throughout her hemodialysis treatment despite high potassium concentrations in the dialysate bath, likely related to kaliuresis from increased aldosterone activity on distal tubules from acetaminophen-induced renal vasoconstriction due to cyclooxygenase (COX) inhibition [24]. However, the possible contributions of the patient's massive ibuprofen ingestion to the presentation of severe metabolic acidosis and kaliuresis cannot be overlooked. In toxic doses of ibuprofen defined as greater than 300 mg/kg, ibuprofen toxicity can lead to severe metabolic acidosis, coma, and renal failure due to the accumulation of acidic metabolites and renal tubular acidosis promoting hypokalemia [25].

## 4. Limitations

Our case report adds to the body of literature suggesting the importance of early consideration of adjunctive therapeutic interventions to NAC in massive acetaminophen toxicity including fomepizole and/or extracorporeal elimination of acetaminophen. However, there are some limitations to acknowledge in this case. Our patient presented with a polypharmacy overdose that included greater than 300 mg/kg of ibuprofen. In addition to the massive acetaminophen overdose, her biochemical high anion gap metabolic acidosis and decreased level of consciousness may also be related to ibuprofen. Ibuprofen is heavily protein bound, and hemodialysis has limited utility in elimination however may help in normalization of acid base abnormalities [26]. Our patient presented with abnormalities that would not be clinically expected in the setting of massive ibuprofen toxicity including a high methemoglobin concentration. This suggests that acetaminophen was the likely predominant contributor to the clinical presentation. Thus, in our case, it cannot be asserted with complete certainty that the combination of triple therapy with NAC, fomepizole, and extracorporeal elimination was superior to a different combination these therapies given the possible contributions of metabolic acidosis from ibuprofen toxicity.

## 5. Conclusion

Our case of a massive acetaminophen overdose demonstrates the early systemic consequences of mitochondrial dysfunction and how rapid and aggressive intervention with NAC, fomepizole, and intermittent hemodialysis can be effective adjunctive therapies in the management of such cases. Despite largely preclinical data in support of the use of fomepizole, appropriate clinical circumstances may warrant its use. Hemodialysis remains an additional adjunctive therapeutic option in severe cases, but clinicians must remain cognisant of the pharmacokinetic alterations in NAC and fomepizole clearance that may occur when utilized.
