# Corrigendum to “Massive Acetaminophen Overdose Treated Successfully with N-Acetylcysteine, Fomepizole, and Hemodialysis”

**DOI:** 10.1155/2021/9797319

**Published:** 2021-11-17

**Authors:** Michael H. Chiu, Natalia Jaworska, Nicholas L. Li, Mark Yarema

**Affiliations:** ^1^Department of Critical Care Medicine, Cumming School of Medicine, University of Calgary, AB, Canada; ^2^Department of Medicine, Division of Nephrology, Cumming School of Medicine, University of Calgary, AB, Canada; ^3^Poison and Drug Information Service, Alberta Health Services, Calgary, AB, Canada; ^4^Department of Emergency Medicine, University of Calgary, Calgary, AB, Canada

In the article titled “Massive Acetaminophen Overdose Treated Successfully with N-Acetylcysteine, Fomepizole, and Hemodialysis” [1], there was an error in Figure 1(a), where the unit of acetaminophen in the *y*-axis should be corrected to “*μ*mol/L.”

The authors confirm that this does not affect the results and conclusions of the article.
